# A computational pipeline for identifying kinetic motifs to aid in the design and improvement of synthetic gene circuits

**DOI:** 10.1186/1471-2105-14-S16-S5

**Published:** 2013-10-22

**Authors:** Austin WT Chiang, Ming-Jing Hwang

**Affiliations:** 1 Bioinformatics Program, Taiwan International Graduate Program, Institute of Information Science, Academia Sinica, Taipei, Taiwan; 2 Institute of BioMedical Informatics, National Yang-Ming University, Taipei, Taiwan; 3 Institute of Biomedical Sciences, Academia Sinica, Taipei, Taiwan

## Abstract

**Background:**

An increasing number of genetic components are available in several depositories of such components to facilitate synthetic biology research, but picking out those that will allow a designed circuit to achieve the specified function still requires multiple cycles of testing. Here, we addressed this problem by developing a computational pipeline to mathematically simulate a gene circuit for a comprehensive range and combination of the kinetic parameters of the biological components that constitute the gene circuit.

**Results:**

We showed that, using a well-studied transcriptional repression cascade as an example, the sets of kinetic parameters that could produce the specified system dynamics of the gene circuit formed clusters of recurrent combinations, referred to as kinetic motifs, which appear to be associated with both the specific topology and specified dynamics of the circuit. Furthermore, the use of the resulting "handbook" of performance-ranked kinetic motifs in finding suitable circuit components was illustrated in two application scenarios.

**Conclusions:**

These results show that the computational pipeline developed here can provide a rational-based guide to aid in the design and improvement of synthetic gene circuits.

## Background

The goal of synthetic biology is to be able to engineer biological processes and to select and put together standardized components according to a design and user-specified function and dynamics [1-5]. To this end, several depositories of biological components have been established [6-12], but the design cycles still rely very heavily on the slow and error-prone process of trying out the parts [13,14].

A fundamental problem is that we still lack clear knowledge of the factors that govern the dynamic behaviours of even the very simple circuits that are motifs of large biological networks [13-18]. Although the structure, or topology, of a biological network may largely dictate its dynamics [19-21], the kinetic parameters (e.g. those that indicate the level of efficiency) of the involved biochemical reactions also play a role [22-27]. Thus, when performing a mathematical simulation to determine which biological components from the depositories should be chosen for a synthetically designed biological circuit with a specified dynamics, one needs to consider not only network topology, but also kinetic parameters.

In this work, we developed a computational pipeline, called Kinetic Motif and Functional Analysis (KMFA), to address this problem. By identifying the set of kinetic parameters required to produce the user-specified dynamic behaviour for a given network topology in both the presence and absence of random perturbations and using statistical analysis to identify recurrent patterns (i.e. motifs) of these kinetic parameters and understand their mechanics, KMFA provides a "handbook" of kinetic motifs in which one can look up a biological components library to choose suitable parts for optimal performance of the designed circuit.

We first demonstrated the utility of KMFA by applying it to a well known synthetic gene circuit of a looped cascade of transcriptional inhibitions built in *Escherichia coli *[28-31]. We showed that, for this 4-gene circuit, only 2,355 (0.6%) of the 390,625 (5^8^) possible combinations of the kinetic parameters could produce the prescribed steady-state concentration of each gene product as the output of the circuit under both perturbed and unperturbed conditions and that these kinetic solutions formed clusters of motifs, which could be ranked according to their relative performance, thus yielding a "handbook" of performance-ranked kinetic motifs that can be used to select library components for the circuit. We illustrated this using two scenarios: the first was to identify faulty/sub-optimal components and replace them with suitable ones to make a non-functional circuit functional, while the second was to improve the performance of an already functional circuit. KMFA is therefore a useful computational tool with a rational design capability to choose circuit components in synthetic biology research.

## Methods

**Figure 1:**
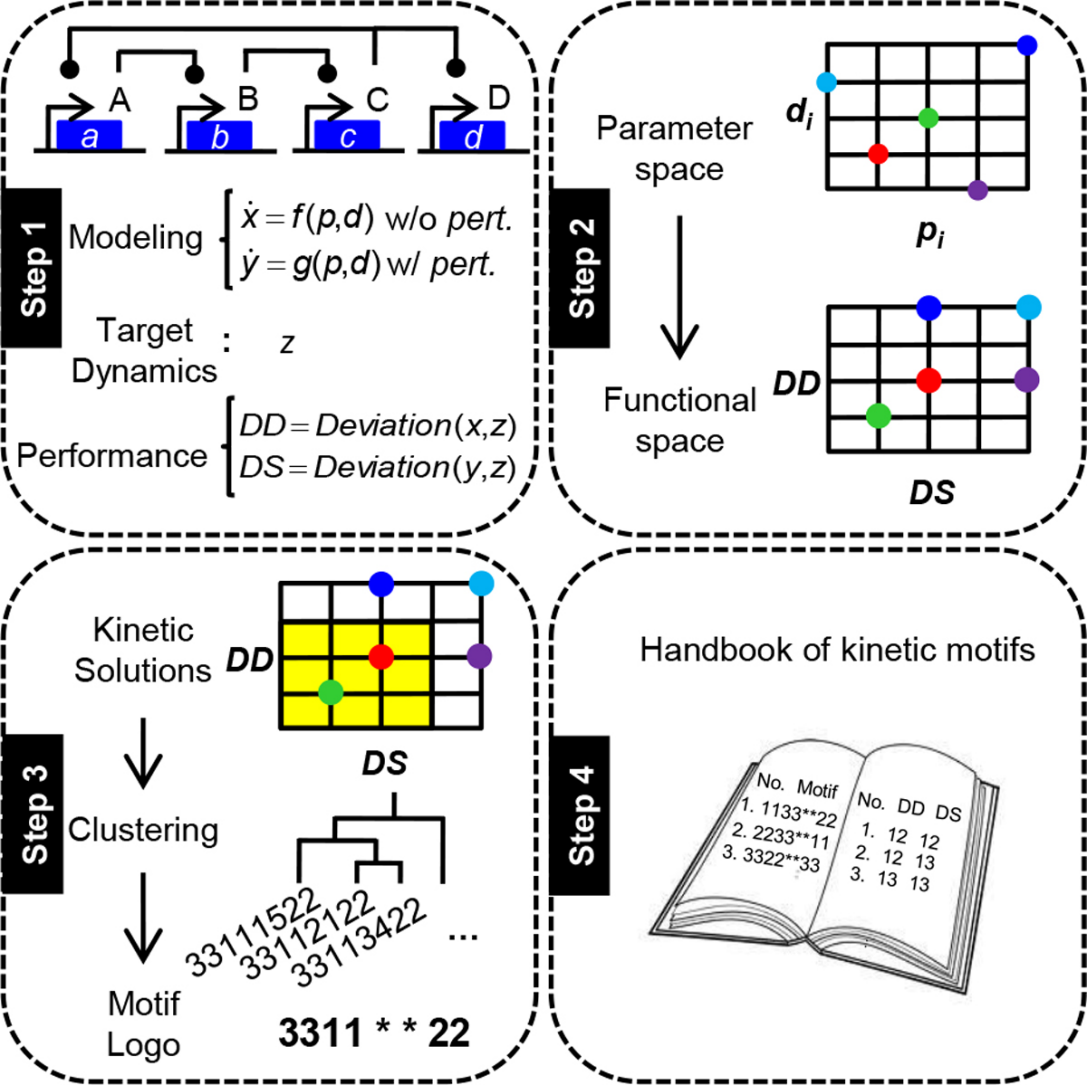
**Steps in the kinetic motif and functional analysis (KMFA) pipeline**. KMFA is a computational pipeline consisting of four steps: system setup, functional mapping by simulation, identification of functional kinetic motifs and tabulation of performance-ranked kinetic motifs. The details of each step are described in the Methods.

As shown in Figure 1, the KMFA pipeline consists of four steps. Although the concept and procedures of KMFA are generally applicable, we used a specific gene circuit to describe these steps and, later, in the Results, illustrate its utilities. This circuit was a four-gene transcriptional cascade that has been synthesized in *E coli *and has been studied both experimentally [28] and computationally [29-31].

### Step 1: System setup

In this step, the system is set up to be numerically simulated in Step 2. This includes specifying the topology of the gene circuit or network, the mathematical model of the system, the target output dynamics and performance measures. Many studies have shown that deterministic modeling and stochastic modeling of biological systems have complementary benefits, the deterministic modeling providing the mean qualitative dynamics of a gene circuit and stochastic modeling the effects of random noises on the circuit [32,33]. In this study, we modeled the dynamics of the transcriptional cascade using both deterministic and stochastic simulations, the latter being used to find kinetic solutions for a robust system that could function properly under perturbations. The mathematical equations for the deterministic simulations are [29]:

(1) $$\begin{array}{ccc} {ẋ}_{i}\left(t\right) & = & f\left(p_{i},d_{i}\right)\\ & = & p_{i,0}+p_{i}r_{i}\left(x_{j}\left(t\right)\right)-d_{i}x_{i}\left(t\right)\\ r_{i}\left(x_{j},t\right) & = & \frac{1}{1+{\left(x_{j}\left(t\right)\;/)\;1000\right)}^{2}} \end{array}$$

where *x_i_*(*t*) denotes the concentration of protein *i *and ${ẋ}_{i}\left(t\right)$ its rate of change at time *t*; *f*(*p_i_*,*d_i_*) denotes the non-linear gene regulation of transcription and translation of protein *i*; *p_i,0 _*is the basal production rate, *p_i _*the production rate constant and *d_i _*the degradation rate constant of protein *i*; and *r_i_*(*x_j_*(*t*)) denotes the Hill function of regulator protein *j *repressing the production of protein *i *at time *t*.

The mathematical equations for the stochastic simulations are [31]:

(2) $$\begin{array}{ccc} {ẏ}_{i}\left(t\right) & = & g\left(p_{i},d_{i}\right)\\ & = & f\left(p_{i},d_{i}\right)+\Delta f\left(p_{i},d_{i}\right)n_{i}\left(t\right)+v_{i}\left(t\right)\\ & = & \left(p_{i,0}+p_{i}r_{i}\left(y_{j}\left(t\right)\right)-d_{i}y_{i}\left(t\right)\right)\\ & \;\;+ & \left(\Delta p_{i,0}+\Delta p_{i}r_{i}\left(y_{j}\left(t\right)\right)-\Delta d_{i}y_{i}\left(t\right)\right)n_{i}\left(t\right)+v_{i}\left(t\right) \end{array}$$

where *y_i_*(*t*) denotes the concentration of protein *i *and ${ẏ}_{i}\left(t\right)$ its rate of change at time *t*; *g*(*p_i_*,*d_i_*) denotes the non-linear gene regulation of transcription and translation of protein *i *influenced by both intrinsic and external random noises; Δ*p*_*i,0*_, Δ*p_i _*and Δ*d_i _*denote the respective standard deviations for the parameters of basal production rate, production rate and degradation rate; and *n_i _*and *v_i _*are, respectively, randomly generated intrinsic and extrinsic noises for protein *i*.

This cascade of transcriptional inhibitions has been shown to produce steady-state concentrations of the proteins involved [28-31]. To facilitate comparison, the target dynamics (*z*) were chosen to be the steady-state concentrations of protein TetR, LacI, CI and Eyfp used in Chen and Wu [31], giving *z *= {1000, 30000, 300, 30000 nM}. To measure the performance of a given set of kinetic parameters (*p_i_*, *d_i_*), *i *= {TetR, LacI, CI, Eyfp}, deviations from the steady-state concentrations of the target were computed for the deterministic simulations using Eq. (3) and for the stochastic simulations using Eq. (4), where *DD *denotes the deterministic deviation, *DS *the stochastic deviation and *ln *a logarithmic transformation function.

(3) $$DD=Deviation\left(x,z\right)=ln\left(\underset{i}{\sum }\underset{t}{\sum }\left|x_{i}\left(t\right)-z_{i}\left(t\right)\right|\right)$$

(4) $$DS=Deviation\left(y,z\right)=ln\left(\underset{i}{\sum }\underset{t}{\sum }\left|y_{i}\left(t\right)-z_{i}\left(t\right)\right|\right)$$

In order to compare with the numerical results of [31], the two measures were not normalized even though *z *varies from 300 to 30000 nM. However, by dictating that a viable dynamics must not deviate from the target concentration by more than 20% for each of the four proteins (see below), we reduced the possibility of the contribution from CI, which has the smallest target concentration at 300 nM, being overtaken by those of the other proteins.

### Step 2: Simulation

For our simulations, we employed the same range of parameter values and values for the basal production rate constants, initial protein concentrations and perturbation fluctuations used by Chen and Wu [31] (summarized in Additional file 1: Table S1). Because it is impossible to enumerate all the real values of the kinetic parameters, we uniformly binned each kinetic parameter into five different rate efficiency levels, with level 1 denoting the lowest and level 5 the highest strength (efficiency) of the kinetics, and used the mean of each bin as the representative of the bin (Additional file 1: Table S2) for the simulations. The five levels may respectively correspond to "weakest", "weak", "medium", "strong", and "strongest" categories that are often used in experimental studies to characterize, say, binding efficiency of ribosome binding sites [7]. Increasing the number of levels will increase the resolution on the transformation of parameter values but will also increase the computational cost and difficulties to identify kinetic motifs in subsequent analysis (see below). Since there are four genes in the system and each gene product (protein) is associated with two kinetic parameters, one for production and the other for degradation, there were a total of 390,625 (5^8^) sets of kinetic parameters that could be used for simulations. For each of these parameter sets, Eq. (1) (deterministic) and Eq. (2) (stochastic) were simulated for a maximum of 100 time steps and differences in protein concentrations between the simulation and prescribed values (*z*) (Eq. (3) and Eq (4)) calculated. At any time point, if the concentration of any of the four proteins exceeded 10^6 ^nM, the simulation (Eq. (1) or Eq. (2)) was aborted prematurely and an extremely large deviation value of 15 was assigned. The other ill-behaved parameter sets, for which a value of 15 was assigned to their *DD *(Eq. (3)) or *DS *(Eq. (4)), were those in which at least one of the four proteins exhibited a steady-state concentration, computed as the mean for the second half of the simulation, that deviated by more than 20% from the prescribed value. Simulations with a deviation value of 15 were considered non-functional.

### Step 3: Identification of kinetic motifs

All the sets of kinetic parameters that were not ill-behaved (i.e. both their *DD *and *DS *values were smaller than 15) were transformed into bin integers representing efficiency levels (see above), which were then clustered hierarchically [34] based on the similarity, computed by the Hamming distance [35], of the sequence of bin levels. This resulted in clusters of kinetic parameters, and each cluster, called a kinetic motif, could be represented by a consensus sequence logo, such as {(1, 1); (3, 3); (*, *); (3, 3)}, in which bin levels in integers of the two kinetic parameters for the production and degradation of each of the four proteins are paired in parentheses and * indicates any levels.

### Step 4: Generation of a handbook of kinetic motifs

These motifs could be ranked based on their performance, i.e. how well they could produce the specified system dynamics, as measured by the deviations *DD *(Eq. (3)) and *DS *(Eq. (4)). This resulted in a table, or handbook, of performance-ranked kinetic parameter sets, which provides information that can be easily referred to in order to identify suitable genetic components to assemble a functional circuit that meets user-desired specifications.

## Results

### Mapping parameters to solutions

**Figure 2:**
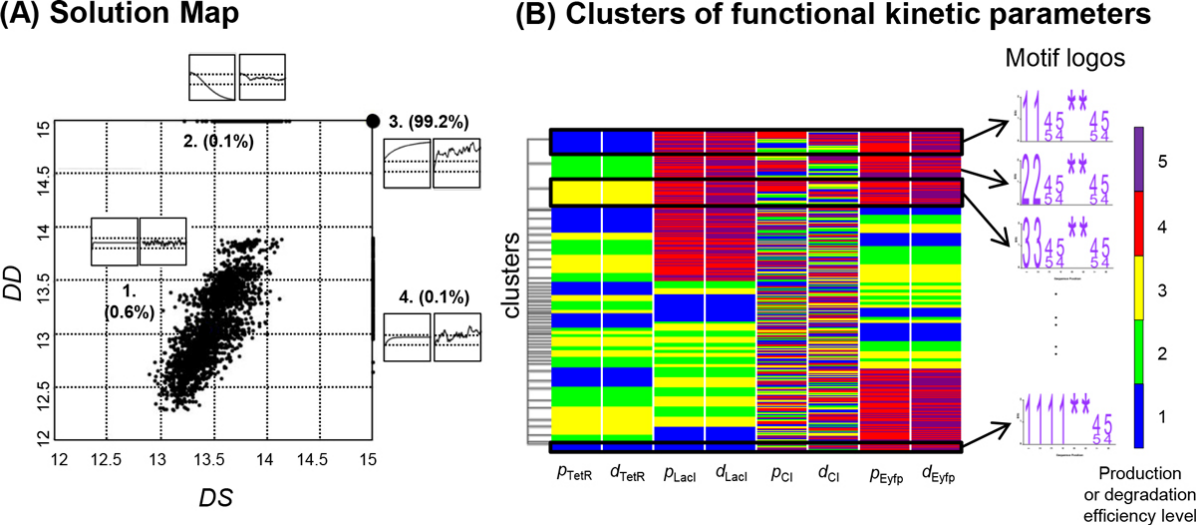
**Identification and clustering of functional kinetic motifs**. (A) The solution map, in which each black dot represents the simulation result, as measured by deviations *DD *and *DS*, for one parameter set and the large black dot in the upper right corner represents many simulation results, because the values of their *DD *and *DS *were both set at 15 to indicate ill-behaved dynamics (see Methods). The resulting dynamics from both the deterministic (i.e. without perturbation) and stochastic (i.e. with perturbation) simulation for the simulated 4-gene transcriptional repression network fell into one of four groups. The vast majority (99.2%) failed to produce the specified dynamics, i.e. the specified steady-state concentration of the protein product of each of the four genes, in either the deterministic or the stochastic simulation, while a very low percentage succeeded in one, but not both, of the two types of simulation (0.1% for each). Those that succeeded in both also accounted for a very small percentage (0.6%) of the kinetic parameters simulated. A typical dynamics is shown for each of the four groups as insets; that on the left is deterministic and that on the right stochastic. (B) Clusters of kinetic motifs. The functional sets (2,355 sets or 0.6% of those sampled) were hierarchically clustered based on the integer sequence of their kinetic efficiency levels, which are colour-coded according to the spectrum shown to the far right, and each cluster could be represented by a motif logo (see Methods).

As described in the Methods, we comprehensively searched the parameter space, albeit using representatives of uniformly divided bins, for sets of kinetics parameters that could produce the specified steady-state system dynamics under both conditions of with and without perturbations. Figure 2A shows that only a very small fraction (2,355 or 0.6%) of the parameter sets sampled (5^8 ^= 390,625) could produce the specified dynamics, while the vast majority (387,535 or 99.2%) failed to function properly under either condition. Interestingly, some parameter sets (0.1%) functioned well only without perturbations, while others (0.1%) did so only with perturbations.

### Clusters of kinetic motifs

Each of the 2,355 kinetic solutions obtained in Figure 2A is a combination of 8 kinetic parameters consisting of the pair of kinetic parameters *p*, for the production rate, and *d*, for the degradation rate of each of the four proteins. The resulting 2,355 8-integer sequences were compared and grouped into 52 clusters (Figure 2B), 48 of which had at least 10 members (i.e. 10 parameter sets that could produce the specified steady-state dynamics). Similar to sequences of DNA [36] or amino acids [37-39], these clusters of kinetic parameter sets, or kinetic motifs, were represented by a motif logo made up of efficiency level integers in which the character size reflects the extent of consensus within the cluster, while '*' indicates no consensus at all (Figure 2B). These 48 clusters were tabulated in a handbook of kinetic motifs (Additional file 1: Table S3), along with their circuit structure, kinetic motif logo, number of cluster members and performance scores.

### Functional association of kinetic motifs

From Additional file 1: Table S3, in which the clusters of kinetic motifs are ranked by their performance in decreasing value of the sum of their *DD *and *DS *scores, we observed that, on the whole, the top clusters tended to have high, though not necessarily the highest, production and degradation rates, i.e. there were more 3's or 4's and 5's than 1's and 2's in the motif logos, and that, conversely, levels 1 and 2 occurred more frequently in the motif logos for the clusters ranked at the bottom. This was particularly true for the 2^nd ^(LacI) and 4^th ^(Eyfp) proteins in the network, because they were required to have a high steady-state output concentration of 30,000 nM, compared to 1,000 nM for the 1^st ^protein (TetR) and 300 nM for the 3^rd ^(CI) (see Methods). Furthermore, for the same protein, its production and degradation rates seemed to be symmetry-related, i.e. level 3 degradation tended to be paired with level 3 production. Such symmetry is also visible in Figure 2B. These observations are consistent with our understanding of the dynamic behaviours of a transcription regulation system in that, in general, degradation rates determine the response time and fast degradation rates allow rapid changes in protein concentration and can, therefore, minimize the response time to stimulation [40,41]. Furthermore, to maintain a given steady state concentration, proteins with a rapid degradation rate also require a high production rate, the final concentration being determined by both rates [16,42]. Inspection of individual solutions with the highest degradation rate (level 5) showed that they tended to exhibit high fluctuations in protein concentrations before reaching the final steady-state concentrations, suggesting that the highest degradation rate (level 5) may not always be the most desirable for the designed circuit, which may also explain why the symmetry between protein production and degradation tended to break down a little when levels were very high (4 or 5; Additional file 1: Table S3).

Another interesting observation was that the third protein (CI) did not show a preference for a particular efficiency level for either of its two kinetic parameters (Figure 2B and Additional file 1: Table S3). As shown in Additional file 1: Fig. S1, this can be explained by examining the repression network and its rate equations. Briefly, the high initial and steady-state concentrations of protein LacI, which represses the *cI *gene, renders the contribution of gene regulation to CI production negligible and, thus, the magnitude of its production kinetic parameter, *p*_CI_, inconsequential; also, as a consequence, at the steady state, all the values within the range allowed for its degradation kinetic parameter, *d*_CI_, would lead to a CI concentration within 20% of the specified value, thus meeting the required condition for a functional solution (see Methods).

### Application scenario I: making a non-functional design functional

**Figure 3:**
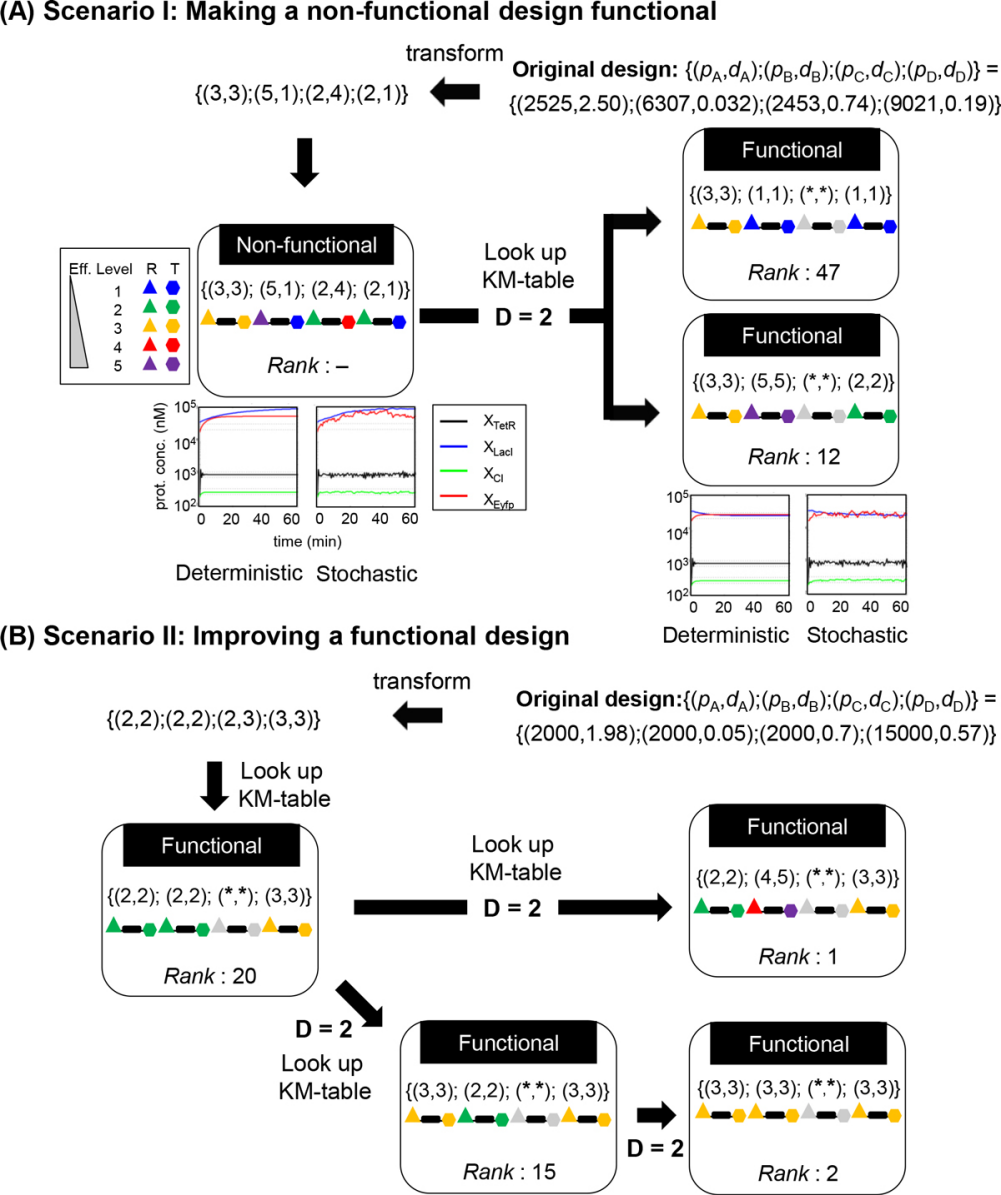
**Two scenarios demonstrating the application of KMFA**. (A) Scenario I: Making a non-functional design functional. If we have a non-functional original design, as indicated by the dynamics of the deterministic and stochastic simulation shown, we can transform its kinetic parameters into efficiency levels and look in the handbook of kinetic motifs (Additional file 1: Table S3) for a similar, but functional, design to replace some of the ribosome binding sites (RBS, denoted as R and represented by triangles) and/or protein degradation tags (PDT, denoted as T and represented by hexagons) with others with a suitable efficiency level to render the modified design functional. In this example, two different routes of minimal changes (Hamming distance (D) of replacement = 2) were found. (B) Scenario II: Improving a functional design. In this example, the original, already functional, design is that reported by Chen and Wu [31], the ranking of which in our performance table (Additional file 1: Table S3) can be improved by selecting components with an RBS and/or PTD that can work with other components to generate a better system performance (i.e. better performance ranking in Additional file 1: Table S3). All notations and symbols are the same as those in (A).

Given a non-functional circuit, it is, at present, not easy to determine the reasons for the failure to function or to identify the faulty/sub-optimal component that needs to be replaced or corrected to salvage the design. The handbook of kinetic motifs and associated properties (Additional file 1: Table S3) is a great aid to solving this problem, as illustrated by the following example. As shown in Figure 3A, the original network was composed of components with kinetic parameters {(*p*_TetR_, *d*_TetR_); (*p*_LacI_, *d*_LacI_); (*p*_CI_, *d*_CI_); (*p*_Eyfp_, *d*_Eyfp_)} with values equivalent to efficiency levels of {(3, 3); (5, 1); (2, 4); (2, 1)} that could not produce the specified dynamics for the 2^nd ^(LacI) and 4^th ^(Eytp) proteins. Comparing this sequence of kinetic levels with those tabulated in Additional file 1: Table S3, we found two motifs with a sequence that differed at only two of the 8 positions (i.e. Hamming distance = 2), namely motif No. 47 [{(3, 3); (1, 1); (*, *); (1, 1)}] and motif No. 12 [{(3, 3); (5, 5); (*, *); (2, 2)}]. Note that, in this comparison, we ignored the 3^rd ^protein (CI) because, as mentioned above, it does not have a preference for these kinetic levels. To modify the original design into one of these two motifs, we can change the efficiency level of the kinetic parameters by using a different ribosome binding site (RBS) for one or more of the genes and thereby changing their protein production level or by using a different protein degradation tag (PDT) to alter the protein degradation rate. Specifically, we can either replace the RBS in the original design with one with a protein production efficiency level of 1 for the 2^nd ^and 4^th ^proteins to change the original design into one that would behave like motif No. 47 or we can replace the PDT in the original design with one with protein degradation efficiency levels of 5 for the 2^nd ^protein and 2 for the 4^th ^protein, making it resemble motif No. 12. Both routes of modification would render the system functional, although the second would lead to a system with a better performance. Note that the key rule in making these changes is to maintain symmetry between the protein production and degradation rates for the same protein, as discussed above.

### Application scenario II: improving a functional design

In the second scenario (Figure 3B), the original circuit was already functional and, in fact, has been shown to be robust under perturbations by Chen and Wu [31]. However, it was ranked in the middle part of Additional file 1: Table S3 (motif No. 20) and therefore could still be improved. There is more than one way of improving the performance of the original design. The two proposed routes of change illustrated in Figure 3B would both lead to a superior performance ranking in the "handbook" of this circuit: the first involves increasing the efficiency level for both the RBS and PDT for the 2^nd ^protein (LacI), while the second requires the two steps of replacing the RBS and PDT for the 1^st ^protein (TetR), then those for the 2^nd ^protein (LacI).

## Discussion

Mathematical modelling and simulations are being increasingly used to help design simple biological circuits to achieve user-specified functions or certain network behaviours in synthetic biology [5,43-47]. Much work has been focused on finding suitable network topologies [19-21], or kinetic parameters that can produce the desired system dynamics, given a network topology [22-26,29-31]. In this work, we have developed a computational pipeline, KMFA, for the latter. Compared to previous studies, a distinctive feature of KMFA is that, by comprehensively mapping the parameter space and functional space (Figure 1, Step 2), the identified multiple sets of functional kinetic parameters can be clustered and their motifs (i.e. their recurring combinations) identified (Figure 2B) and analysed to reveal and understand significant functional associations (see Results, Figure 2).

In this work, the network was simulated with and without perturbations, the former being modeled by a stochastic process with randomly introduced intrinsic and extrinsic noises (Eq. (2)) and the latter by a deterministic process (Eq. (1)). The results showed that there are certain situations (i.e. certain combinations of the kinetic parameters) in which the system may function well under perturbations, but not in the absence of perturbations. Although it is well recognized that noise is an integral part of normal biological functions and how biological systems evolve to be robust [48-53], our results suggest that, to design a 'truly robust' biological system, both conditions (with and without noise) need to be considered. Although further analysis is required to elucidate the mechanism for the effect of perturbation observed in this study, we speculate that perturbation alters the "background" protein production and degradation rates to allow the circuit to achieve and maintain target dynamics. Indeed, several studies have shown that gene circuits (e.g., switches and oscillations) rely on noise to achieve desired functions and would fail without them [54-57].

As a proof-of-principle study, KMFA has been designed in the present work to exhaust all possible kinetic parameter combinations, which limits its application to very small systems such as the transcriptional cascade serving as a demonstrating example above. However, this limitation can be lifted by integrating with approaches such as Monte-Carlo sampling [58], Latin hypercube sampling [59], and others [29,60] that have been developed to efficiently search for parameter solutions from high dimensional spaces. Indeed, several large systems (e.g. the Fas apoptotic pathway [61]) have been studied using some of these methods. Replacing our time-consuming simulation step (see Figure 1) with these efficient parameter-searching methods, one can collect the parameter solutions for an interesting function/dynamics of a large system, and then apply KMFA on those parameter solutions to find kinetic motifs.

An important finding of this study is that, as in studies of network topologies [19-21], only a very small percentage of kinetic parameters can be functional, i.e. produce the desired system dynamics (Figure 2A). Obviously, network topology and kinetic parameters are intertwined, and the kinetic motifs (Additional file 1: Table S3) identified here will probably be specific not only for the specific arrangement of the components of the circuit, but also for the specified output protein concentrations. Nevertheless, for a given circuit design, the "handbook" of kinetic motifs (Additional file 1: Table S3) will be very useful in deciding which biological components available from a biological parts library, such as BioFab [10] and the MIT Registry of Standard Biological Parts [7], should be used, as illustrated by the two application scenarios presented in Figure 3. Thus, the handbook could be used to standardize the biological parts (e.g. by classifying them into 5 efficiency levels) and, via ranking all possible combinations of the parts, standardize the circuit design process. The handbook could furthermore be used to help synthesize required parts of a specified efficiency level not yet collected in the library, especially the ribosome binding sites for which a relationship between binding efficiency and binding site sequence has been elucidated [62]. As more and more genetic components (e.g. promoters, ribosome binding sites, terminators and protein degradation tags) are deposited and characterized, mathematical simulations using a computational pipeline, such as KMFA, will make the synthetic design and engineering of biological circuits more efficient and also more rationale-based. Furthermore, the general applicability of the KMFA approach will allow simulations of different network topologies and different functions (see Additional file 1 for additional illustration on an 'AND'-gate circuit [63,64]) in comparative studies to uncover intriguing common/distinct principles of biological networks, particularly those pertaining to kinetic parameters, which have received less attention than network topologies.

## Conclusions

Knowledge about how to choose suitable components for a designed gene circuit is required for efficient research in synthetic biology. For a given topology of a designed gene circuit, the computational pipeline, KMFA, developed here, has produced a "handbook" of performance-ranked kinetic motifs that can serve as a user guide to allow the selection and matching up of different genetic components to achieve user-specified system functions. In addition to being a useful aid for the synthetic design of biological circuits, KMFA can also be used to elucidate the intertwined relationship of the trinity of prototype biological circuits: topology, kinetics of the parts and function.

## Competing interests

The authors declare that they have no competing interests.

## Authors' contributions

Conceived and designed the experiments: AWTC MJH. Performed the experiments: AWTC. Analyzed the data: AWTC MJH. Wrote the paper: AWTC MJH.

## Supplementary Material

Additional file 1 **Table S1**: Ranges and values of parameters used for simulations in this study. **Table S2**: RBSs and PDTs of different efficiency levels used in this study for the structural arrangement RBS-gene-PDT. **Table S3**: Handbook of kinetic motifs for the transcriptional repression cascade circuit simulated. **Figure S1**: Analytical analysis of the kinetic parameters of protein CI. **Additional illustration**: The case of an 'AND'-gate circuit. Click here for file

## Declarations

The publication costs for this article were funded by Academia Sinica, Taipei, Taiwan.

This article has been published as part of *BMC Bioinformatics *Volume 14 Supplement 16, 2013: Twelfth International Conference on Bioinformatics (InCoB2013): Bioinformatics. The full contents of the supplement are available online at http://www.biomedcentral.com/bmcbioinformatics/supplements/14/S16.
