# Supplementary material for: A computational pipeline for identifying kinetic motifs to aid in the design and improvement of synthetic gene circuits
Source: BMC Bioinformatics. 2013 Oct 22;14(Suppl 16):S5. doi: 10.1186/1471-2105-14-S16-S5 (PMC3853143; doi:10.1186/1471-2105-14-S16-S5)
Supplement: Additional file 1 — Table S1: Ranges and values of parameters used for simulations in this study. Table S2: RBSs and PDTs of different efficiency levels used in this study for the structural arrangement RBS-gene-PDT. Table S3: Handbook of kinetic motifs for the transcriptional repression cascade circuit simulated. Figure S1: Analytical analysis of the kinetic parameters of protein CI. Additional illustration: The case of an 'AND'-gate circuit. [file 1471-2105-14-S16-S5-S1.PDF]

Supplementary Data

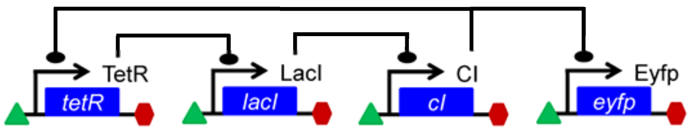

$$r_{CI}(x_{LacI}) = \frac{1}{1+(x_{LacI}/1000)^2} = \frac{1}{1+(30000/1000)^2} \approx 0.001$$

$$\frac{dx_{CI}}{dt} = p_{CI,0} + p_{CI} * r_{CI}(x_{LacI}) - d_{CI} * x_{CI} = 210 + \boxed{p_{CI} * 0.001} - d_{CI} * x_{CI} \approx 210 - d_{CI} * x_{CI}$$

1) compared to 210, p_{CI} * 0.001 is generally negligible.

At steadystate:

$$x_{CI} \approx \frac{210}{d_{CI}}$$

$$(1-20\%) * 300 \le x_{CI} \le (1+20\%) * 300$$

$$\frac{7}{8} \ge d_{CI} \ge \frac{7}{12}$$

$$\boxed{0.875 \ge d_{CI} \ge 0.583}$$

2) the original range of d_{CI} is [0.6 – 0.8](Table S1), which is within this inequality, so d_{CI} is unconstrained.

p_{CI} and d_{CI} are unconstrained.

Figure S1 - Analytical analysis of the kinetic parameters of protein CI.

At the steady state (x_{LacI} = 30,000 nM), the repression of cI by LacI is very strong (Hill coefficient = 0.001). Thus, any production rate (p_{CI}) for the *cI* gene (Table S1) will result in negligible production of CI protein, explaining the finding that p_{CI} did not exhibit a preference for a particular efficiency level. Also, as a result, the steady-state concentration of CI protein is $210/d_{\mathrm{CI}}$. Because x_{CI} is specified in the inequality: $(1 - 20\%) * z \leqq x_{\mathrm{CI}} \leqq (1 + 20\%) * z$ (z = 300 nM, see Methods and Table S1), the substitution of $210/d_{\mathrm{CI}}$ yielded $0.583 \leqq d_{\mathrm{CI}} \leqq 0.875$. This derived range of d_{CI} covers all degradation rates of CI ($0.6 \leqq d_{\mathrm{CI}} \leqq 0.8$) provided in Table S1, which explains why d_{CI} also did not exhibit any preference for a particular efficiency level.

Table S1 – Ranges and values of parameters used for simulations in this study

Parameters	TetR	LacI	CI	Eyfp
p_0 (nM • min^{-1})	150	587	210	3487
p (nM • min^{-1})	50 ~ 5000	70 ~ 75000	75 ~ 8000	30 ~ 30000
d (min^{-1})	0.2 ~ 4.8	0.02 ~ 0.14	0.6 ~ 0.8	0.1 ~ 1.0
$\triangle p_0$ (nM • min^{-1})	30	50	30	50
$\triangle p$ (nM • min^{-1})	50	200	50	200
$\triangle d$ (min^{-1})	0.3	0.3	0.3	0.3
$x(0)$ (nM)	200	40000	200	20000
z (nM)	1000	30000	300	30000

These parameter values were obtained from Chen and Wu (B. S. Chen and C. H. Wu, *BMC Systems Biology*, 2009, **3**, 66).

Table S2 – RBSs and PDTs of different efficiency levels used in this study for the structural arrangement RBS-gene-PDT

Efficiency Level	RBS-gene	Production rate (nM • min^{-1})				gene-PDT	Degradation rate (min^{-1})			
		p_{TetR}	p_{LacI}	p_{CI}	p_{Eyfp}		d_{TetR}	d_{LacI}	d_{CI}	d_{Eyfp}
1	R_1()	545	763	868	3027	T_1()	0.66	0.032	0.62	0.19
2	R_2()	1535	2149	2453	9021	T_2()	1.58	0.056	0.66	0.37
3	R_3()	2525	3535	4038	15015	T_3()	2.50	0.080	0.70	0.55
4	R_4()	3515	4921	5623	21009	T_4()	3.42	0.104	0.74	0.73
5	R_5()	4505	6307	7208	27003	T_5()	4.34	0.128	0.78	0.91

A triangle represents the ribosome binding site (RBS), a rectangle the corresponding gene and a hexagon the protein degradation tag (PDT) of its gene product. The colour scheme for the kinetic efficiency level is the same as that in Fig. 2B and Fig. 3 in the main text: blue for level 1, green for level 2, yellow for level 3, red for level 4 and purple for level 5.

Table S3 – Handbook of kinetic motifs for the transcriptional repression cascade circuit simulated

Rank	Motif	Circuit	Members	*DD*	*DS*	*DD+DS*
1	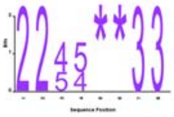	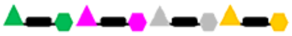	64	13.34	12.80	26.14
2	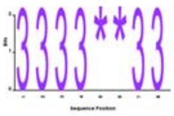	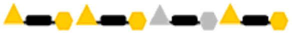	25	13.37	12.79	26.16
3	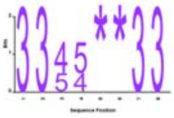	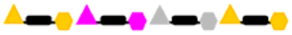	70	13.34	12.84	26.18
4	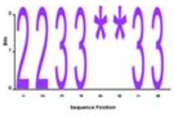	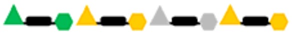	25	13.40	12.80	26.20
5	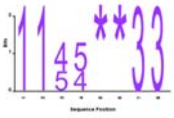	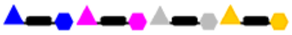	62	13.38	12.86	26.24
6	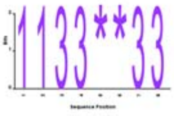	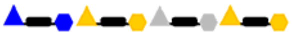	25	13.38	12.88	26.26
7	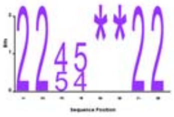	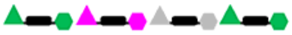	63	13.42	12.90	26.33
8	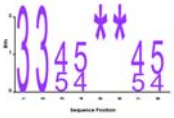	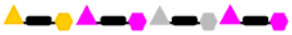	193	13.37	12.97	26.34
9	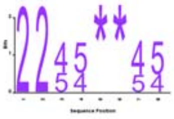	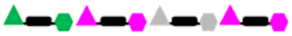	188	13.37	12.97	26.34
10	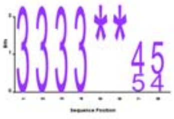	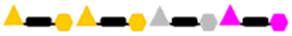	71	13.40	12.94	26.35

The notations are the same as those used in Figs. 1 and 2 in the main text and in Table S2.

Rank	Motif	Circuit	Members	*DD*	*DS*	*DD+DS*
11	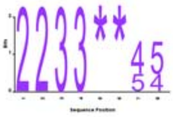	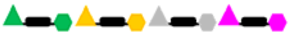	72	13.39	12.96	26.35
12	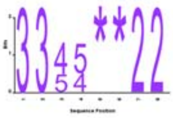	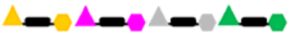	64	13.43	12.92	26.36
13	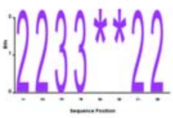	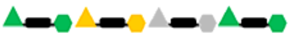	25	13.45	12.91	26.36
14	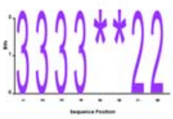	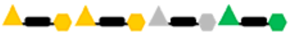	25	13.46	12.90	26.36
15	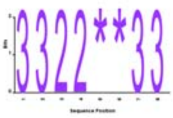	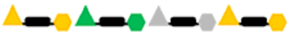	25	13.45	13.01	26.46
16	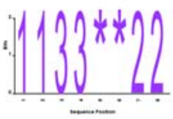	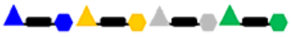	24	13.49	12.98	26.47
17	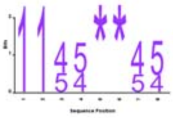	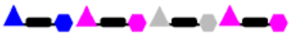	188	13.43	13.04	26.47
18	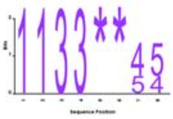	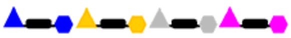	73	13.44	13.04	26.48
19	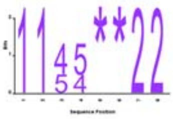	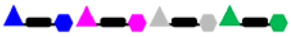	66	13.50	13.00	26.50
20	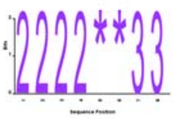	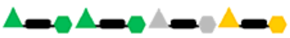	25	13.50	13.02	26.52

Rank	Motif	Circuit	Members	*DD*	*DS*	*DD+DS*
21	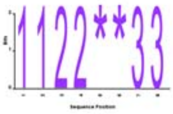	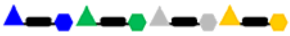	25	13.50	13.07	26.57
22	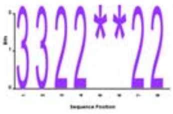	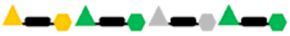	25	13.52	13.10	26.62
23	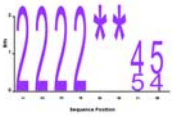	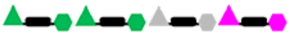	73	13.48	13.16	26.64
24	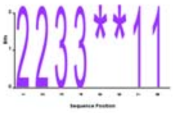	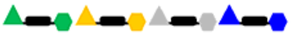	22	13.51	13.13	26.65
25	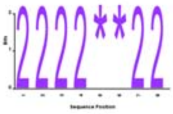	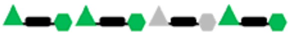	25	13.55	13.10	26.65
26	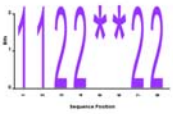	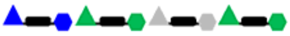	24	13.51	13.16	26.66
27	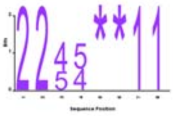	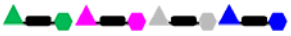	46	13.56	13.11	26.67
28	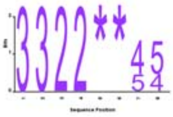	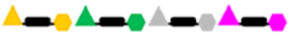	73	13.51	13.17	26.68
29	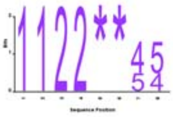	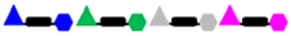	69	13.54	13.19	26.73
30	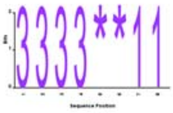	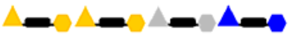	20	13.61	13.13	26.73

Rank	Motif	Circuit	Members	*DD*	*DS*	*DD+DS*
31	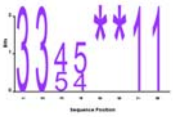	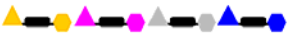	53	13.60	13.15	26.75
32	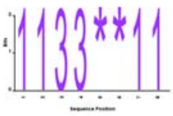	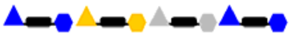	24	13.59	13.19	26.78
33	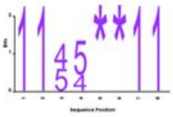	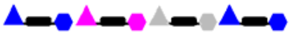	53	13.64	13.19	26.82
34	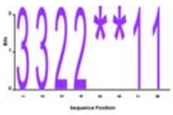	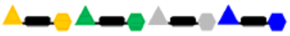	25	13.62	13.29	26.90
35	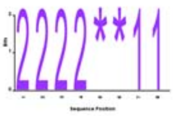	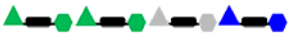	20	13.62	13.29	26.91
36	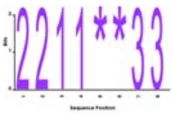	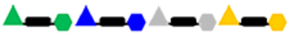	25	13.60	13.35	26.95
37	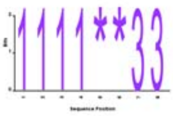	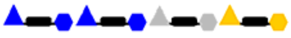	24	13.61	13.37	26.98
38	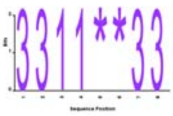	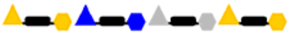	20	13.64	13.35	26.99
39	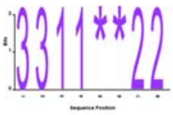	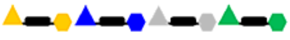	24	13.63	13.41	27.04
40	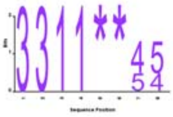	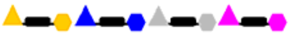	62	13.60	13.45	27.05

Rank	Motif	Circuit	Members	*DD*	*DS*	*DD+DS*
41	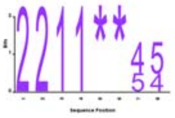	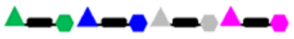	64	13.61	13.44	27.05
42	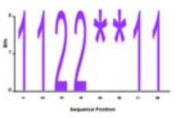	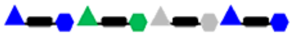	18	13.74	13.34	27.07
43	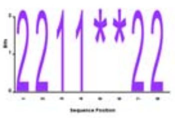	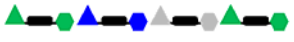	20	13.66	13.41	27.08
44	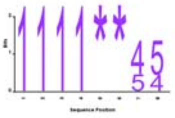	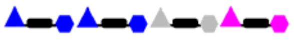	63	13.66	13.50	27.16
45	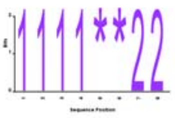	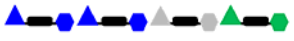	22	13.73	13.44	27.17
46	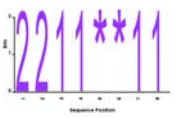	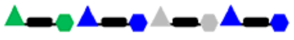	20	13.69	13.55	27.24
47	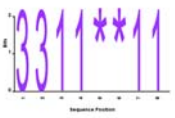	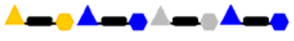	22	13.73	13.55	27.29
48	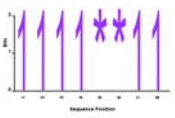	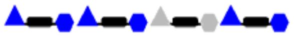	17	13.82	13.58	27.40

Additional illustration - the case of an 'AND'-gate circuit

In this section, we demonstrate the utility of KMFA on an additional example: an 'AND'-gate circuit (Fig. S2). This circuit has been shown to produce system dynamics resembling an 'AND' gate (Fig. S2A). Fig S2B illustrates the gene network of this circuit that has been studied both experimentally [63] and computationally [64]. The input signal I_1 (light) alone can trigger an artificial early stop codon embedded *T7* gene to transcribe a T7 mRNA, and then translate it into a truncated T7 RNA polymerase. The input signal I_2 (IPTG) can trigger the transcription of a *tRNA* gene to prevent the premature termination of T7 RNA polymerase and lead to the translation of a full length (functional) T7 RNA polymerase. The functional T7 RNA polymerase will eventually trigger the GFP express. Briefly, a high concentration of GFP can be detected only if both external input signals (I_1 and I_2) are present. The mathematical equations for the dynamics of this circuit are depticed in Fig. S2C (deterministic) and Fig. S2D (stochastic).

To facilitate comparison, we employed the same parameter values and perturbation fluctuations used in Chen et al. [64] (summarized in Table S4). Values of the kinetic parameters, (k_i, p_i), $i = \{1, 2, 3\} = \{\text{T7, tRNA, GFP}\}$, were binned into five different rate efficience levels, as shown in Table S5. The target dynamics (z) was chosen to be the concentration of protein GFP, at $z = 2000$ (μM) when both of the input signals I_1 and I_2 were present, and at $z = 200$ (μM) otherwise. The performance measures *DD* (deviation in deterministic simulation) and *DS* (deviation in stochastic simulation) for a given set of kinetic parameters $\{(k_1, p_1); (k_2, p_2); (k_3, p_3)\}$ were computed using Eq. (3) and Eq. (4) (see Methods in the main text).

The same analysis procedures as described in the main text were followed. A total of 5^6 sets of parameter values were simulated and we found that, as before, only a very small fraction (446 or 2.85%) of them could produce the specified dynamics. These 446 kinetic solutions were grouped into 16 clusters (see Methods in main text, and Fig. S3). Interestingly, we observed that the 446 solutions all contained three specific combinations of kinetic parameters $\{(k_3, p_3)\}$ relating to GFP (the subscript 3 represents GFP) with values equivalent to efficiency levels of {(1, 5)}, {(2, 2)} and {(5, 1)}. Furthermore, the product of the two parameter values (k_3 and p_3) for the three combinations were all around 0.1 (0.10 * 1.00 =0.10; 0.90 * 0.12 = 0.30 * 0.36 = 0.108), which may suggest that a balanced synergistic interaction between the two kinetic components of GFP is required for the 'AND'-gate dynamics.

Next, as before, KMFA produced a handbook of kinetic motifs for this model (see Table S6), which can be used to select biological components from parts library for desired dynamics and performance. Finally, Fig. S4 showed the simulation results using two parameter sets, one from Chen et al. [64] ({(0.6042, 0.9272); (0.8410, 0.00001); (0.8640, 0.1235)}), and the other being one of many KMFA solutions ({(1.00, 0.30); (0.80, 0.01); (0.30, 0.36)}), where the parameter sets were presented in the order of $\{(k_1, p_1); (k_2, p_2); (k_3, p_3)\}$. The set of parameters values from Chen et al. [64] can be transformed into a set of efficiency levels {(3, 5); (4, 1); (5, 1)}, which belongs to the 1st motif of the handbook (Table S6), while the compared KMFA set exhibited the 3rd motif.

A. Truth table

I_1	I_2	Out (GFP)
0	0	0
0	1	0
1	0	0
1	1	1

B. Model

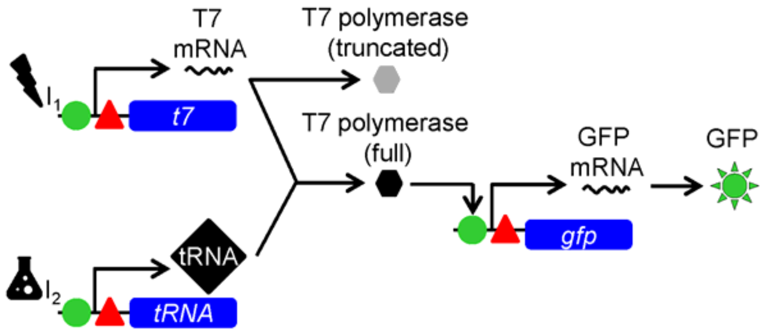

C. Deterministic equations

$$\begin{cases}\frac{dx_i}{dt}=f_x(k_i,d_i)\\ \frac{dy_j}{dt}=f_y(p_j,\lambda_j)\end{cases}$$

$$=\begin{cases}\frac{dx_{T7}}{dt}=k_1I_1-d_1x_{T7}\\ \frac{dx_{tRNA}}{dt}=k_2I_2-d_2x_{tRNA}\\ \frac{dy_{T7^*}}{dt}=p_1x_{T7}-\lambda_1y_{T7^*}\\ \frac{dy_{T7}}{dt}=p_2x_{tRNA}x_{T7}-\lambda_2y_{T7}\\ \frac{dx_{GFP}}{dt}=c+k_3r(y_{T7})-d_3x_{GFP}\\ \frac{dy_{GFP}}{dt}=p_3x_{GFP}-\lambda_3y_{GFP}\\ r(y_{T7})=\frac{(y_{T7}/K)^n}{1+(y_{T7}/K)^n}\end{cases}$$

D. Stochastic equations

$$\begin{cases}\frac{dx_i}{dt}=g_x(k_i,d_i)\\ \frac{dy_j}{dt}=g_y(p_j,\lambda_j)\end{cases}$$

$$=\begin{cases}\frac{dx_{T7}}{dt}=(k_1+\Delta k_1n_1)I_1-(d_1+\Delta d_1n_4)x_{T7}+v_1\\ \frac{dx_{tRNA}}{dt}=(k_2+\Delta k_2n_1)I_2-(d_2+\Delta d_2n_4)x_{tRNA}+v_2\\ \frac{dy_{T7^*}}{dt}=(p_1+\Delta p_1n_2)x_{T7}-(\lambda_1+\Delta\lambda_1n_4)y_{T7^*}+v_3\\ \frac{dy_{T7}}{dt}=(p_2+\Delta p_2n_2)x_{T7}x_{tRNA}-(\lambda_2+\Delta\lambda_2n_4)y_{T7}+v_4\\ \frac{dx_{GFP}}{dt}=c+(k_3+\Delta k_3n_3)r(y_{T7})-(d_3+\Delta d_3n_4)x_{GFP}+v_5\\ \frac{dy_{GFP}}{dt}=(p_3+\Delta p_3n_2)x_{GFP}-(\lambda_3+\Delta\lambda_3n_4)y_{GFP}+v_6\end{cases}$$

Figure S2 - Truth table, model and mathematical equations of the 'AND'-gate circuit studied.

(A) A truth table of the 'AND' circuit: 'I_1' and 'I_2' denote the external input signals, and 'Out' (GFP concentration) denotes the output of the system responding to the input signals. Briefly, the 'AND' circuit would output high GFP concentration only if both input signals were present. (B) Network topology of the 'AND'-gate circuit. (C) Mathematical equations for the deterministic simulation of this circuit [64], where x_i and y_j denote the concentration of mRNA i and protein j, respectively; i = {1, 2 ,3} =

{T7 mRNA, tRNA, GFP mRNA}, and j = {1, 2 ,3} = {T7* polymerase (truncated) , T7 polymerase (full-length), GFP protein}; $f_x(k_i, d_i)$ denotes the transcription of mRNA i and $f_y(p_j, \lambda_j)$ the translation of protein j; I_1 and I_2 denote two different external input signals (light and IPTG); c is the basal transcription rate of GFP mRNA; k is the transcription rate constant and d the degradation rate constant; p is the translation rate constant and λ the degradation rate constant; $r(y_m)$ denotes the non-linear function of regulator protein m activating the transcription of GFP gene. (D) Mathematical equations for the stochastic simulation of this circuit [64], where $g_x(k_i, d_i)$ denotes the perturbation of the transcription of mRNA i and $g_y(p_j, \lambda_j)$ denotes the perturbation of the translation of protein j, where the perturbation is influenced by both intrinsic and external random noises; Δk_i denotes the standard deviation for the parameters of transcription rate, Δd_i that of mRNA degradation rate, Δp_j that of translation rate and $\Delta\lambda_j$ that of protein degradation rate; finally, n_i and v_i are, respectively, randomly generated intrinsic and extrinsic noises.

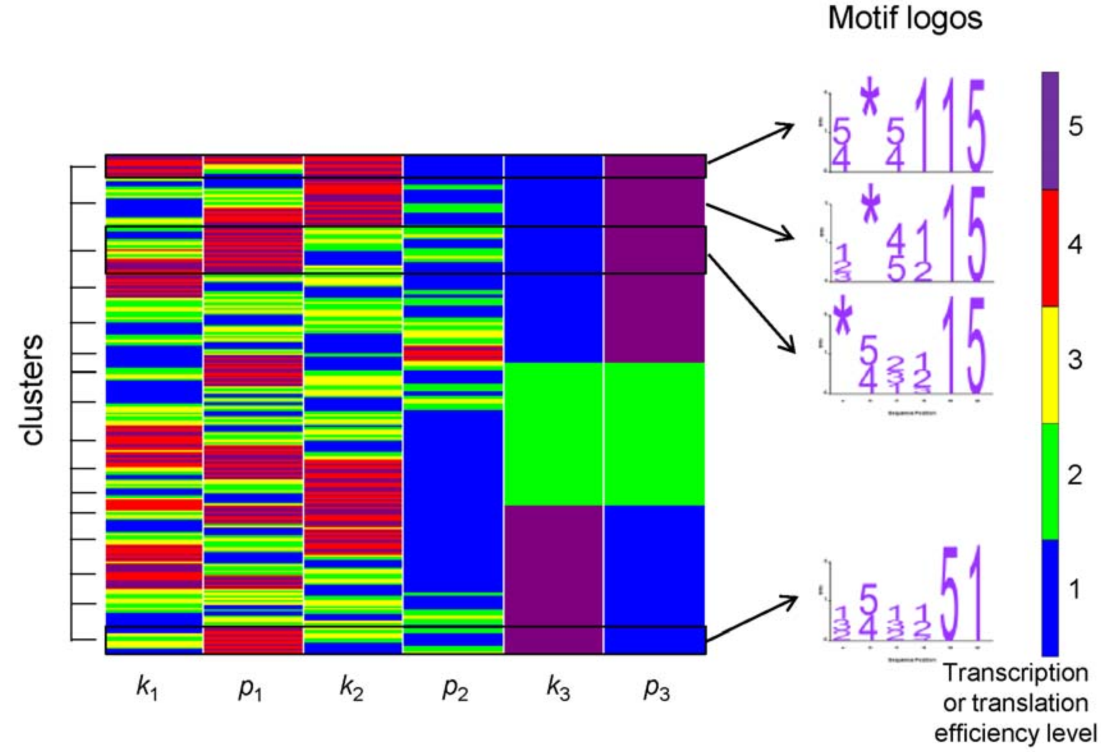

Figure S3 - Clusters of functional kinetic motifs

The functional sets (446 sets or 2.85% of total 5^6 sampled) were hierarchically clustered based on the integer sequence of their kinetic efficiency levels, which are colour-coded according to the spectrum shown to the far right, and each cluster could be represented by a motif logo (see Methods in the main text).

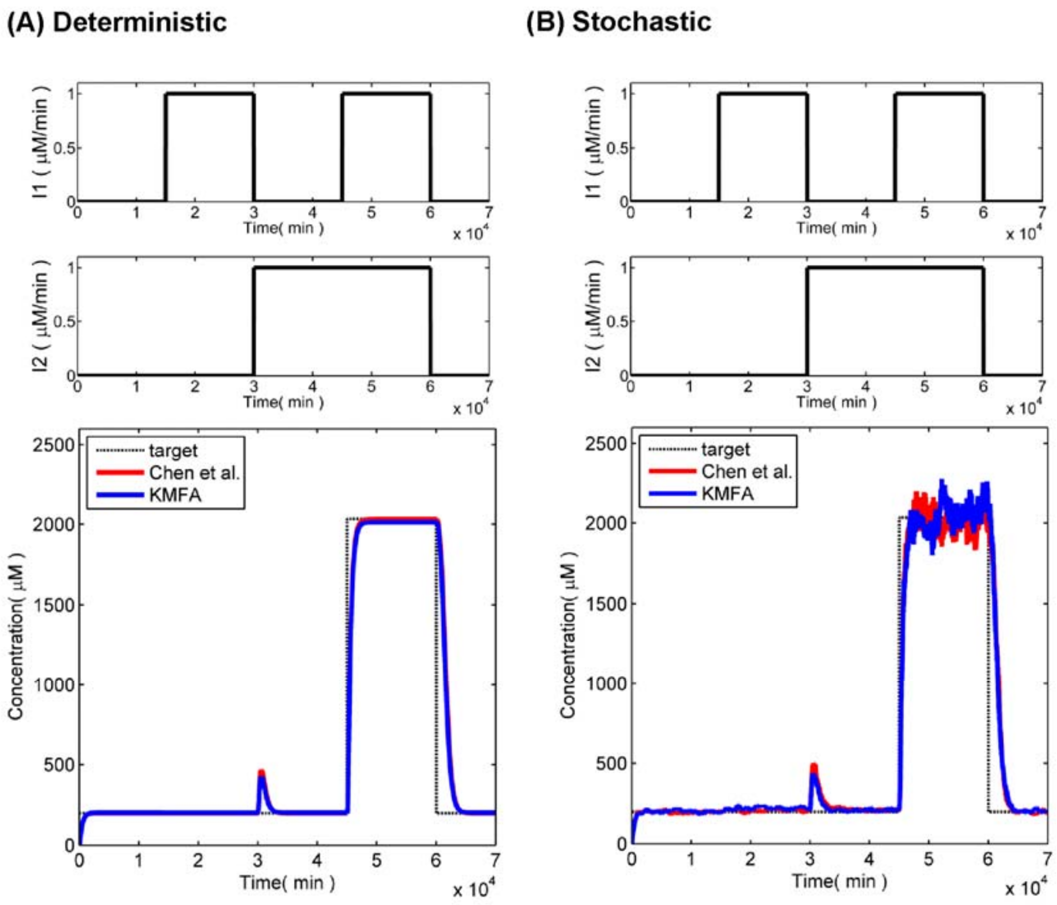

Figure S4 - Simulation results for the 'AND'-gate circuit studied

(A) Deterministic (i.e. without perturbation) and (B) stochastic (i.e. with perturbation) simulation of the 'AND'-gate circuit. The two external input signals (I_1 and I_2) treated with time were specified in the top panel and the middle panel, respectively. The bottom panel shows the resulting dynamics for this circuit using two parameter sets derived from Chen et al. (red) and KMFA (blue), in comparison to the target dynamics (the black dashed line). The parameter set from Chen et al. is {(k_1, p_1); (k_2, p_2); (k_3, p_3)} = {(0.6042, 0.9272); (0.8410, 0.00001); (0.8640, 0.1235)}, and from KMFA is {(1.00, 0.30); (0.80, 0.01); (0.30, 0.36)}. The peaks at time tick 3 were a result of an effect of a time delay of I_1.

Table S4 – Ranges and values of parameters used for simulations in this study

Parameters	1 (T7)	2 (tRNA)	3 (GFP)
k (-)	0 ~ 1	0 ~ 1	0 ~ 1
p (min^{-1})	0 ~ 1	0 ~ 0.1	0 ~ 1
d ($\mu M \cdot min^{-1}$)	0.0231	0.0231	0.0231
λ ($\mu M \cdot min^{-1}$)	0.0023	0.0023	0.0023
Δk (-)	0.2	0.2	0.2
Δp (min^{-1})	0.02	0.000005	0.02
Δd (-)	0.04	0.04	0.04
$\Delta \lambda$ (min^{-1})	0.05	0.05	0.05

Note: '-' denotes dimensionless. These parameter values were obtained from Chen et al. (Chen et al., *Journal of Biomedicine and Biotechnology*, 2011:304236).

Table S5 – Promoters and RBSs of different efficiency levels used in this study for the structural arrangement Promoter-RBS-gene

Efficiency Level	Promoter-RBS-gene	k (-)			Promoter-RBS-gene	p (min^{-1})		
		k_1	k_2	k_3		p_1	p_2	p_3
1	P_1(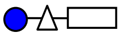)	0.20	0.20	0.10	R_1(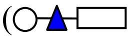)	0.10	0.01	0.12
2	P_2(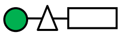)	0.40	0.40	0.30	R_2(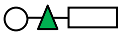)	0.30	0.03	0.36
3	P_3(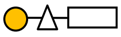)	0.60	0.60	0.50	R_3(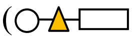)	0.50	0.05	0.60
4	P_4(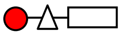)	0.80	0.80	0.70	R_4(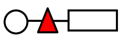)	0.70	0.07	0.84
5	P_5(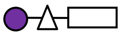)	1.00	1.00	0.90	R_5(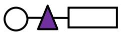)	0.90	0.09	1.00

Note: '-' denotes dimensionless; a circle represents the promoter, a triangle the ribosome binding site (RBS) and a rectangle the corresponding gene. The colour scheme for the kinetic efficiency level is the same as that in Fig. S3: blue for level 1, green for level 2, yellow for level 3, red for level 4 and purple for level 5.

Table S6 – Handbook of kinetic motifs for the ‘AND’-gate circuit studied

Rank	Motif	Circuit	Members	*DD*	*DS*	*DD+DS*
1	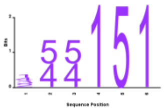	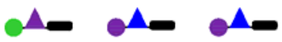	16	17.89	18.13	36.02
2	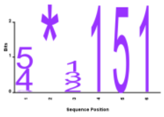	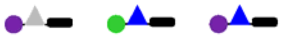	31	17.90	18.13	36.03
3	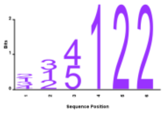	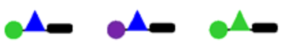	23	17.89	18.15	36.04
4	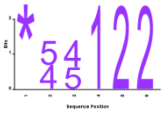	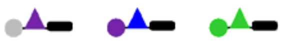	18	17.90	18.15	36.06
5	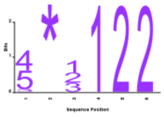	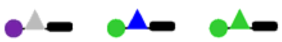	31	17.92	18.15	36.07
6	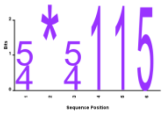	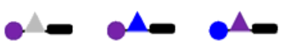	20	17.98	18.13	36.10
7	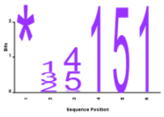	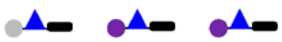	28	17.92	18.18	36.09
8	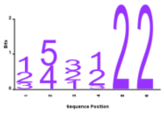	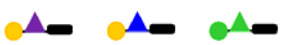	21	18.01	18.17	36.19
9	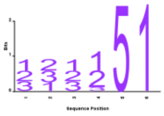	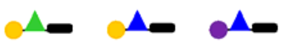	33	18.00	18.19	36.19
10	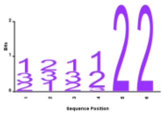	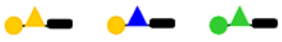	34	18.03	18.19	36.22

The notations are the same as those used in Fig. 1 in the main text and in Table S5.

Rank	Motif	Circuit	Members	*DD*	*DS*	*DD+DS*
11	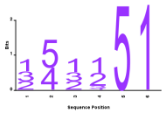	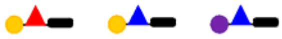	25	18.03	18.20	36.23
12	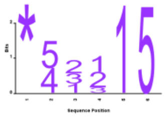	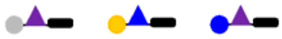	42	18.12	18.22	36.34
13	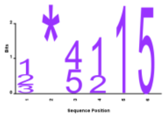	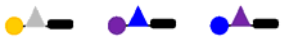	44	18.12	18.22	36.34
14	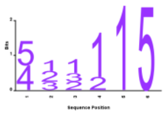	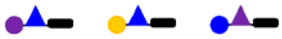	22	18.13	18.22	36.35
15	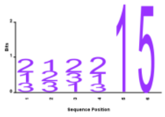	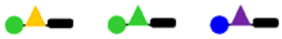	43	18.15	18.26	36.41
16	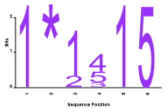	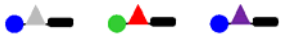	15	18.14	18.29	36.43
